# Homeostatic Regulation of Pro-Angiogenic and Anti-Angiogenic Proteins via Hedgehog, Notch Grid, and Ephrin Signaling in Tibial Dyschondroplasia

**DOI:** 10.3390/ani13243750

**Published:** 2023-12-05

**Authors:** Shah Nawaz, Muhammad Fakhar-e-Alam Kulyar, Quan Mo, Wangyuan Yao, Mudassar Iqbal, Jiakui Li

**Affiliations:** 1 College of Veterinary Medicine, Huazhong Agricultural University, Wuhan 430070, China; malikshahnawaz786@gmail.com (S.N.); fakharealam786@hotmail.com (M.F.-e.-A.K.); wangyuay@ucr.edu (W.Y.); mudassar.iqbal@iub.edu.pk (M.I.); 2 Department of Animal Nutrition and Feed Science, College of Animal Science and Technology, Huazhong Agricultural University, Wuhan 430070, China; 3 Faculty of Veterinary and Animal Sciences, The Islamia University of Bahawalpur, Bahawalpur 63100, Pakistan

**Keywords:** angiogenesis, pro-angiogenic proteins, anti-angiogenic proteins, tibial dyschondroplasia, bone, chondrogenesis

## Abstract

**Simple Summary:**

Tibial dyschondroplasia (TD) is an avian metabolic disease characterized by rapid apoptosis and reduced angiogenesis with compromised chondrocyte activity at the growth plate and, ultimately, poor osteogenesis. Thiram, a widely recognized fungicide used to preserve fruits and grains, is considered among one of the potential predisposing factors of TD through sonic hedgehog, notch-gridlock, and ephrin-B2/EphB4 pathways of angiogenesis. Current literature provides a comprehensive overview regarding the involvement of pro and anti-angiogenic proteins in the mechanism of TD development.

**Abstract:**

Precise coupling of two fundamental mechanisms, chondrogenesis and osteogenesis via angiogenesis, plays a crucial role during rapid proliferation of growth plates, and alteration in their balance might lead to pathogenic conditions. Tibial dyschondroplasia (TD) is characterized by an avascular, non-mineralized, jade-white “cartilaginous wedge” with impaired endochondral ossification and chondrocyte proliferation at the proximal end of a tibial bone in rapidly growing poultry birds. Developing vascular structures are dynamic with cartilage growth and are regulated through homeostatic balance among pro and anti-angiogenic proteins and cytokines. Pro-angiogenic factors involves a wide spectrum of multifactorial mitogens, such as vascular endothelial growth factors (VEGF), platelet-derived growth factors (PDGF), basic fibroblast growth factor (bFGF), placental growth factors, transforming growth factor-β (TGF-β), and TNF-α. Considering their regulatory role via the sonic hedgehog, notch-gridlock, and ephrin-B2/EphB4 pathways and inhibition through anti-angiogenic proteins like angiostatin, endostatin, decoy receptors, vasoinhibin, thrombospondin, PEX, and troponin, their possible role in persisting inflammatory conditions like TD was studied in the current literature review. Balanced apoptosis and angiogenesis are vital for physiological bone growth. Any homeostatic imbalance among apoptotic, angiogenetic, pro-angiogenic, or anti-angiogenic proteins ultimately leads to pathological bone conditions like TD and osteoarthritis. The current review might substantiate solid grounds for developing innovative therapeutics for diseases governed by the disproportion of angiogenesis and anti-angiogenesis proteins.

## 1. Introduction

Bone is a connective tissue with a substantial blood supply. Vasculature is essential for bone development and regeneration since it is the primary source of nutrition, oxygen, immune cells, mesenchymal stem cells, hormones, neurotransmitters, and growth factors transportation for bone cells [1]. Bone tissue development is a complicated mechanism involving osteogenic and angiogenic mechanisms, which can promote bone proliferation and tissue regeneration [2]. Blood vessel development is essential to bone formation, skeletal development, and the osseo-integration process. It plays a crucial role in the transportation of growth factors to enable cell survival, interaction, and angiogenesis in different tissue regeneration vessels in the bone marrow beside the growth plate. It also exemplifies the central element of a metabolically unique bone microenvironment with advantageous access to nutrients and oxygen by improving the growth potential of adjacent perivascular cells, for example, pre-osteoprogenitors, osteoprogenitors, and mature and hypertrophic chondrocytes [3,4]. Both organ growth and tissue healing depend on forming a functional circulatory system. Blood arteries in bone provide calcium and phosphate, the components for mineralization, in addition to oxygen and nutrition. Furthermore, it appears that the bone marrow’s blood arteries play a significant role by acting as a habitat for both bone-forming skeletal stem cells and blood-forming hematopoietic stem cells [5].

Tibial dyschondroplasia (TD) is the primary clinical disease caused by various factors, like vitamin D deficiency, higher calcium–phosphorus ratio, high growth rate, *Fusarium* mycotoxins, genetic factors, and dithiocarbamates (thiram) exposure to wild and poultry birds [6,7], characterized by impaired endochondral ossification and chondrocyte proliferation. It creates an avascular, unmineralized, jade-white “cartilaginous wedge” that impairs production and induces lameness [8]. Therefore, it has a significant adverse effect on the economic performance of the poultry sector. Previous studies found that TD is the cause of almost 30% of chicken bone illnesses and has a prevalence of more than 10% in China [9]. According to the findings of various studies, the two primary elements contributing to TD development are the lack of angiogenesis and the inability of chondrocytes to calcify [10,11]. The frequency of TD on farms is usually difficult to accurately determine. In fact, broilers with TD have weak legs, restricted behavior, and even difficulty in walking. Broilers are more prone to suffer fractures during the feeding process, which has a detrimental effect on the wellbeing of the birds and lowers productivity, costing the poultry sector significantly. The pathogenesis and prevention of TD have been the constant focus of numerous studies worldwide [12,13]. In the majority of cases, the origin of the disease has been linked to factors related to nutrition, the environment, and genetics [14,15]. Other parameters such as feeding density, dietary elements (such as the ratio of electrolytes, calcium, and phosphorus), vitamin D3, and toxins may also have a role (thiram in particular) [9,16,17,18]. The underlying causes of tibial dyschondroplasia are numerous and appear to be multifactorial. For instance, aberrant levels of biochemical markers like IL-1 and nitric oxide and other variables, including deficiency of vitamin D, hyperthyroidism, and soybean meal in feed, have been linked with fluctuations in the incidence of TD [19,20]. In addition, according to the findings of certain studies, metabolic acidosis, copper deficiency, fusarochromanone, high dietary levels of cysteine, and homocysteine exposure are important risk factors [21]. Reduced chondrocyte propagation and differentiation have been associated with TD, which is connected to the abnormal ossification and proliferation of tibial growth plates [22]. A perfect blood supply and adequate mineralization are essential components of the cartilage matrix, but this is not always the case with TD [23]. TD’s hypertrophic zone cells are the hallmark of the disease, characterized by nuclear absence, deterioration, disarranged columns, and growth plates with deficient angiogenesis [24].

Pesticide residues in feed are becoming an important concern due to their significant global rise in recent decades [25]. Between them, thiram is a member of the dithiocarbamate class of pesticides and a byproduct of various other pesticides (Ferbam, Ziram) that are widely used in agriculture as a pesticide and fungicide to treat cereals intended for seeding and persists in the environment [26,27]. Thiram is deleterious to poultry and acts as a predisposing factor for TD [15]. Food contaminated with thiram residues is hazardous to human and animal health and the environment [28]. These residues produce TD by triggering apoptosis, reducing chondrocyte papulation and vascularization. Because of their lipophilic nature, their mode of action involves inducing cytotoxicity upon adhering to the cell membrane, damaging cartilage cells, disrupting endochondral bone formation, and suppressing angiogenesis. Immune suppression and other negative effects on chickens have also been linked to thiram exposure [29]. Multiple studies demonstrate that the mitochondrial destruction of chondrocytes is triggered by thiram-induced TD (50 mg/kg) via the Cytochrome-C/Bax/Caspase-3 pathway, accelerating apoptosis [30]. Thiram exposure has the potential to affect the development and maturation of chondrocytes by activating the JAK/STAT signaling system and suppressing the BMP/Smad pathway, IHH/PTHrP signaling, and the AKT/PI3K pathway [31].

TD is also associated with angiogenesis because bones require nutrients to grow, and disruption of angiogenesis during cartilage formation prevents bone growth, ultimately resulting in damage to the tibial growth plate known as TD [32]. As tibial growth plate (TGP) lesions encourage calcification of the hypertrophic cartilage matrix and create a bone marrow cavity, blood vessels are crucial for the proper development of avian bone. Additionally, TD in broilers could be caused by inadequate circulation in the TGP. The number of blood vessels for TD chickens’ tibiotarsus bone showed reduced TGP hypertrophic zones. Thiram induces TD by reducing the expression of chicken genes linked to angiogenesis. Additionally, there is an association between chondrocytes and angiogenesis [2]. A remarkable reduction in blood vessel distribution was also found in the TGP. So, it could also be hypothesized that the reduction in erythrocytes is also associated with TD in broiler chickens [32,33]. Moreover, molecular studies demonstrate that the TD could be caused by the vascular endothelial growth factor (VEGF) pathway due to the considerable divergence of critical genes. TD is linked to downregulating genes involved in bone vascularization, mineralization, and other hereditary variables [19,34]. Apoptosis, an aberrant form of cell death, is also connected to the potential pathophysiology of TD in growth plates. The growth plate’s limited vascularity prevents the swift elimination of these deceased chondrocytes. The development of TD results from the expression of the immune system and angiogenesis-related gene associated with chicken erythrocytes [32]. Based on the studies mentioned above, it is possible to post ulate that angiogenesis is impacted by various circumstances, which reduces the flow of nutrients to the growth plate and inhibits bone formation. Apoptosis may be the factor affecting chondrocytes and angiogenesis, leading to the accumulation of an opaque avascular mass at the growth plate that makes it difficult for birds to move appropriately. All the relevant studies on TD indicates a strong correlation between angiogenesis and chondrocytes. So, the following paper was designed to provide a comprehensive review regarding the cellular mechanism of TD as a result of environmental thiram pollution.

### 1.1. Endochondral Ossification and Growth Plate Generation

**Figure 1:**
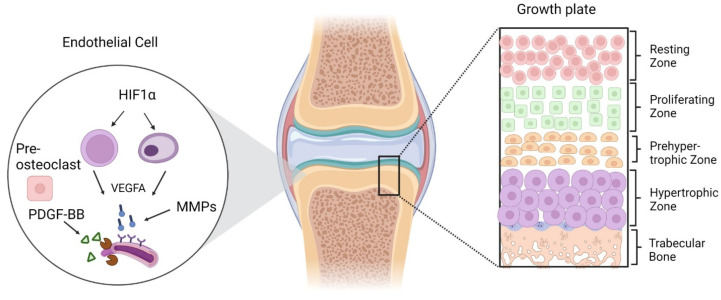
Zonal illustration of the growth plate and endothelial cells signaling through different secretory factors for angiogenesis and ultimate bone growth. The formation of blood vessels initiated by HIF1α activation and further regulation of VEGFA, MMPs, and some other secretory factors from pre-osteoclasts.

The proliferation rate of cartilaginous cells in the growth plate is directly correlated with the longitudinal growth rate of the bone. Endochondral ossification begins with the condensation of loosely congregated, fibroblast-like mesenchymal stem cells (MSCs) [35,36]. Before developing into chondrocytes, this condensation process involves cell-to-cell contact, resulting in areas of high cell density. For cartilage production, chondrocytes release and put together a complex extracellular matrix (ECM) that contains type II collagen and proteoglycans like aggrecan. This cartilage anlage serves as an initial blueprint for the formation and development of bones. These cartilage templates are transformed into bone by endochondral ossification. As endochondral ossification progresses, chondrocytes gradually grow larger [35,37,38,39], characterized by the synthesis of type X collagen and volumetric swelling. These hypertrophic chondrocytes stimulate the bone collar’s development by enhancing the mineralization of the surrounding ECM, releasing vascular endothelial growth factor to direct blood vessels into adjacent ECM, and inducing mineralization of the surrounding ECM. Mineralized cartilage is replaced by bone through the coordinated elimination of hypertrophic chondrocytes via cell death as well as the recruitment of osteoblasts and osteoclasts. Osteoblasts from the bone collar and the main spongiosa (the originally created bone) develop into cortical and trabecular bone as the bone expands. The end of the bone develops a secondary ossification center, and the space between the secondary ossification and the primary spongiosa is known as the growth plate (Figure 1) [40].

From the secondary to the primary ossification center, the growth plate maintains a multi-layered chondrocyte structure, starting with the resting zone, which contains a pool of less differentiated chondroblasts and is also characterized by a low proliferation rate, proteoglycan, and collagen-type II production. The zone where chondroblasts proliferate and differentiate further is known as the proliferative zone and is characterized by the mitotic division of cells. There has been a dramatic increase in collagen production in the actual germinal layer between type II and XI. The enlarged area becomes calcified, degrades, and is eventually replaced by bone. Beneath proliferative zone is the transformation zone, which is subdivided into an upper and lower hypertrophic as well as a degenerative zone. In this zone, chondrocytes can be identified by their lack of cellular proliferation and reduced DNA synthesis. In the line of erosion, osteoclasts and chondroclasts begin to replace the cartilage’s calcifying matrix, and in the primary ossification zone, osteoblasts and osteoclasts communicate to form the primary spongiosa [38,41,42].

### 1.2. Cartilaginous Tibial Dyschondroplasia

**Figure 2:**
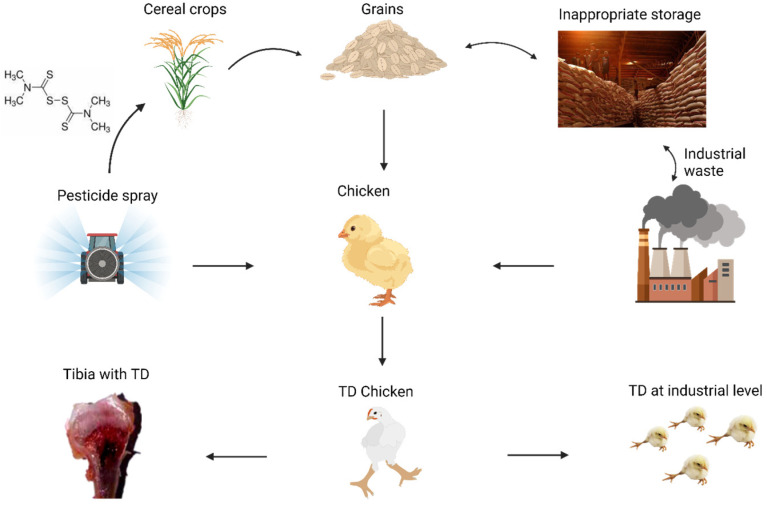
Primary factors involved in the development of TD. These include pesticides (thiram), inadequate feed storage, and industrial waste.

The growth plate controls ossification by maintaining a balance between chicken chondrocyte proliferation and differentiation [43]. Thiram and other environmental pollutants released by commercial industries are thought to be the main cause of TD. Thiram damages the cartilage cells, interferes with the production of endochondral bone, and prevents the growth of new blood vessels by adhering to the cell membrane [29]. Furthermore, molecular research showed that TD may be prompted by the vascular endothelial growth factor (VEGF) pathway due to the substantial divergence of critical genes. TD is linked to genes that affect bone vascularization, mineralization-related genetic variables, and suppression of angiogenesis (Figure 2) [33].

### 1.3. Pathogenic Cellular Factors and Their Pathways in TD

Researchers may establish a successful therapeutic plan to cure disease by looking into the mechanism of disease emergence and understanding all the cellular and extracellular elements involved through their own distinct pathways. Two biological mechanisms, apoptosis and angiogenesis, are thought to play a major role in the etiology of tibial dyschondroplasia. The current investigation focuses mostly on the angiogenesis-stimulated pathways of TD pathogenesis.

### 1.4. Angiogenesis and Related Cellular Proteins

Angiogenesis is a complicated and well-orchestrated process that is dependent on extensive signaling networks both among and within endothelial cells (ECs), their accompanying mural cells (vascular smooth muscle cells (VSMCs), and pericytes, as well as other cell types (e.g., immune cells). These cells are known collectively as endothelial cells. There is a wide range of elements involved in angiogenesis (Table 1).

**Figure 3:**
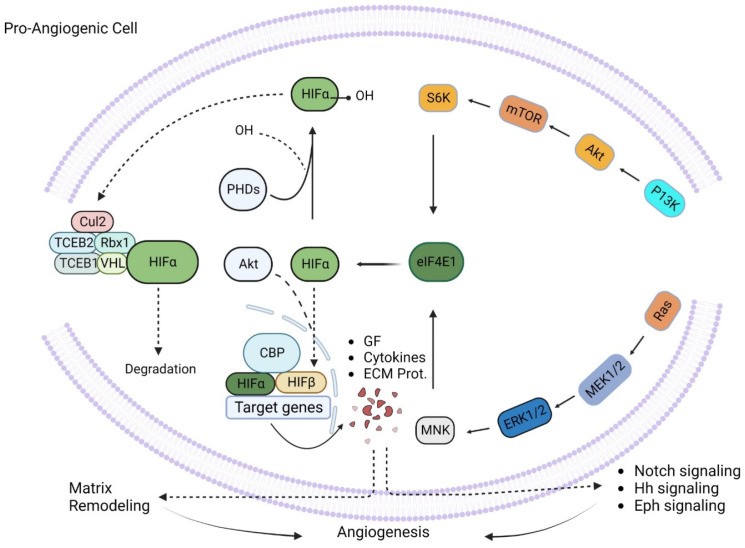
Pro-angiogenic signaling. Pro-angiogenic factors like P13K and Ras initiate a cascade of signaling processes through eIF4E1 and HIFα and cause upregulation of different growth factors, cytokines, and extracellular matrix proteins, which ultimately promote matrix remodeling and hence nurture angiogenesis.

Angiogenesis mandates a group of proteins known as vascular endothelial growth factors (VEGF) [44]. Various VEGF isoforms, including VEGF-A, B, C, and VEGF-D, each play a significant role throughout different angiogenic events ranging from lymphatic to embryonic angiogenesis. The main regulator of angiogenesis is VEGF-A [45]. Four primary VEGF-A isoforms with varied length, 121, 165, 189, and 206 amino acids, are produced by alternative splicing. These isoforms have a variable affinity for heparan sulfate proteoglycans (HSPG). A pioneering tip cell, an endothelial cell that responds to angiogenic signaling, is produced when the ratio of freely diffusible to HSPG-bound VEGF-A is balanced. Through a series of cell migration phases, the tip cells develops the leading edge of the angiogenic sprout and vascular branching [46]. The notch-gridlock, ephrin-B2/EphB4, and sonic hedgehog (SHH) pathways are involved in the division of the branches into arteries, capillaries, veins, and lymphatic vessels when they first expand in response to site-specific metabolic demands. Platelet-derived growth factor (PDGF-B) is secreted by ECs in response to hemodynamic changes and additional vascular development, attracting pericytes and VSMCs [47,48,49]. These mural cells attach to Ecs by expressing angiopoietin-1 (ANG-1), which activates TGF and causes the deposition of the extracellular matrix (ECM), stabilizing the expanding vascular bed. Downstream effectors, including phosphatidylinositol-3 kinase (PI3K), Src kinase, focal adhesion kinase (FAK), p38 mitogen-activated protein kinase (p38 MAPK), Smad2/3, and phospholipase C gamma (PLCγ)/Erk1/2, promote EC survival, vascular permeability, and migratory/proliferative characteristics. Postnatal angiogenesis is also influenced by positive and negative transcriptional control of these moieties by microRNAs (miRNAs) (Figure 3). It has been demonstrated that miR-126 plays an extremely important role because its loss results in defective vascular development and embryonic mortality [49,50].

Similarities between pathological and physiological angiogenesis in terms of signaling processes and the subsequent changes in cell function and behavior suggest that they may represent innovative therapeutic approaches for treating such diseases. However, the formation of abnormal blood vessels does not stop when normal blood flow returns to the affected area. The development of new angiogenesis-disrupting drugs is hampered by such uncontrolled and unresolved growth.

## 2. HIF-1α/VEGF/VEGFR Signaling Pathways under Thiram Exposure

An in vitro study revealed that thiram exposure increases methemoglobin (MetHb) levels in erythrocytes, aggravates reactive oxygen (ROS) and nitrogen species production, reduces marker enzymes of glycolysis (hexokinase, glyceraldehyde 3-phosphate dehydrogenase, pyruvate kinase), and hence decreases NADPH levels, which has adverse effects on antioxidant enzymes like glyoxylase. In this way, thiram inhibited erythrocyte metabolic pathways and caused hemolysis. Thiram-treated cells also had more osmotically fragile activities of acetylcholinesterase with the inhibition of Na+/K+ ATPase-. The increased MetHb level decreases erythrocytes’ oxygen-carrying capacity and hence leads to hypoxia. Morphological changes in thiram-treated erythrocytes alter their rheological properties and lower their flexibility and ability to pass through micro-capillaries [51], which could be among the potential causes of less angiogenesis in GP.

Experimental exposure to thiram causes downregulation of HIF-1α, VEGFA, VEGFR, and β-catenin expressions and upregulation of GSK-3β expression [52,53]. VEGF produced by hypertrophic chondrocytes [54], and regulated by HIF-1α [55]. The capillary invasion-mediated VEGF has an important role during chondrogenesis and osteogenesis via angiogenesis [34]. In contrast, GSK-3β is activated in cellular stress conditions and may lead to cellular death [56]. At the same time, the proliferation, differentiation, and angiogenesis of chondrocytes were inhibited, which may be due to the direct effect of thiram on chondrocytes. In vitro studies showed similar results regarding both gene and protein expression of HIF-1α, VEGFA, and β-catenin. Thiram supplementation (50 mg/kg) in feed significantly reduced the mesodermic differentiation marker gene (FGF2 and FGFR1) level in GP and hence a remarkable reduction in vasculogenesis was observed [57]. Apoptosis-related mediators like Bcl-2/Bax/Caspases-3 and CD147 were found in higher concentrations in the GP of thiram-induced TD birds [30,58]. Additionally, it was found that BH3-only proteins activate Bax and Bak proteins of chondrocytes after thiram treatment and accelerate cytochrome-c production through increased mitochondrial membrane-associated potential, caspase 9, caspase 3, and ultimately apoptosis [30]. All of the above findings confirm the potential role of thiram in TD pathogenesis via imbalanced apoptosis and angiogenesis.

### 2.1. Sonic Hedgehog Pathway

**Figure 4:**
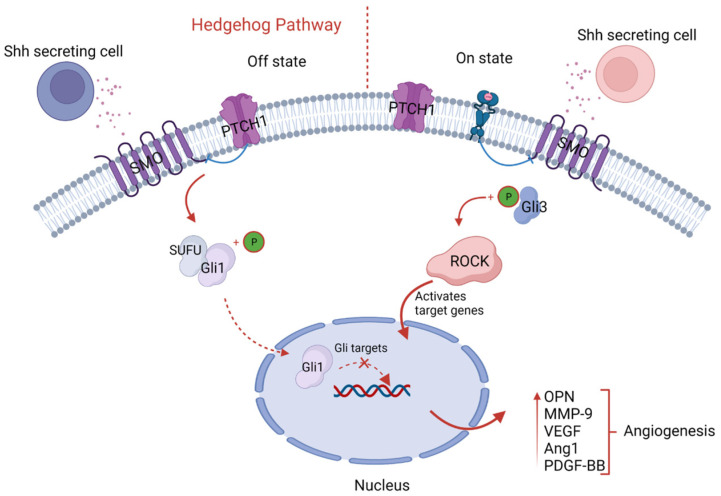
Schematic illustration of hedgehog signaling. The sonic hedgehog cells’ secretion binds with membrane receptors, Patched (Ptch1), and sequentially causes upregulation of Gli target genes through binding with intracytoplasmic proteins ROCK and Gli3, which results in upregulation of pro-angiogenic factors like OPN, MMP-9, VEGF, and angiotensin-1, fostering angiogenesis.

During embryonic development, hedgehog (Hh) proteins such as sonic hedgehog (Shh), Indian hedgehog (Ihh), and desert hedgehog function as morphogens in many tissues. Hedgehog signaling is required for bone formation because it controls chondrocyte proliferation and differentiation [59]. Researchers have focused on Ihh’s ability to promote chondrocyte proliferation, specify bone-forming osteoblasts, and regulate chondrocyte differentiation via a negative feedback loop with parathyroid hormone-related protein (PTHrP). In this context, Gli transcription factors are the primary mediators of hedgehog signaling. In contrast to Gli1, Gli2, and Gli3, which jointly mediate biological activities of hedgehog signaling in osteoblast specification, Gli3 plays a predominant role in growth plate chondrocytes [60,61]. The sonic hedgehog (Shh) morphogen pathway stimulates pro-angiogenic cytokines’ expression in adults, promoting neovascularization [62]. ROCK controls cell polarity and migration by stimulating cell contractions, protrusions, and focal adhesions. By increasing OPN and MMP-9 expressions and stimulating Rho/ROCK-mediated enhancement of VEGF, Ang1, and PDGF-BB, Shh enhances EC migration and vascular development and, ultimately, intensifies angiogenesis. [63]. When Hh-antagonists were administered for only two days, the results of the experimental study were consistent, suggesting that sporadic or familial mutations in the Hh pathway genes or any other abnormality lead to insufficiency of the hypertrophic zone of the growth plate itself to promote mineral deposition and angiogenesis [64]. However, growth plate mineralization was restored once the Hh-antagonist was removed, indicating a potential function for the Hh-mediated route in the development of TD (Figure 4).

### 2.2. Notch-Gridlock Pathway

**Figure 5:**
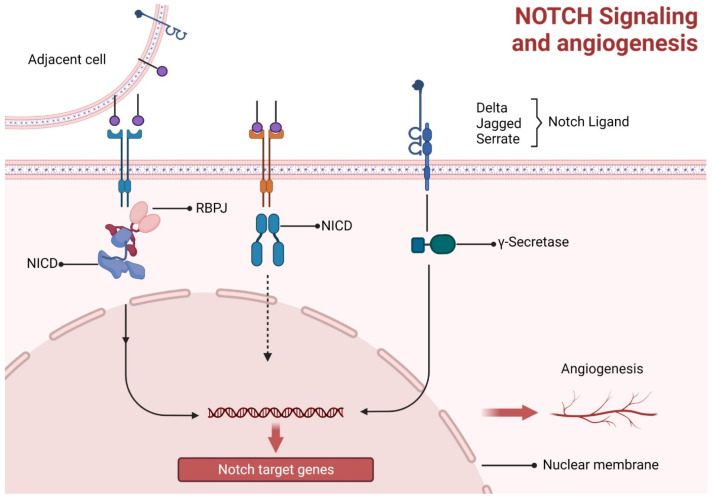
Notch signaling and angiogenesis. Notch ligand signaling stimulates intracytoplasmic immunoglobulin kappa J region (RBPJ) and NICD interaction, while in the nucleus, NICD functions as a transcriptional coactivator for RBPJ and hence angiogenesis is initiated by their interaction.

There are numerous processes by which angiogenesis takes place. Sprouting angiogenesis is one of these processes. This mechanism necessitates coordinating numerous cellular processes and is initiated by liberating various cellular growth factors. Deliberate migrating tip cell selection at the top of the sprout, a well-balanced ratio between newly developing endothelial cells (Ecs), and the conservation of prevailing vascular tubes are three crucial processes [65,66]. According to studies, notch signaling interacts with the notch ligands and communicates with other signaling pathways, such as VEGF signaling, to play a crucial role in angiogenesis. It has been demonstrated that the protein known as delta-like ligand 4 (DLL4) is an important notch ligand that stimulates angiogenesis [65,67,68,69]. When a notch receptor binds with a notch ligand extracellularly, the receptor’s proteolytic cleavage is triggered, kicking off the canonical notch pathway. Secretase and disintegrin metalloproteinase domain-containing protein 10 (ADAM10) are required for the subsequent proteolytic processes following this cleavage. After the initial contact between the notch receptor and ligand, a cascade of cleavage events occur, expulsing the membranous notch receptor intracellular domain (NICD). In the nucleus, NICD functions as a transcriptional coactivator through an association with the recombination of signal-binding protein for the immunoglobulin kappa J region (RBPJ). Notch target gene transcription controls EndoMT and angiogenesis is Initiated by NICD-RBPJ interaction (Figure 5) [70].

Moreover, DLL4’s competitor, Jagged1 (Jag1), can negatively regulate angiogenesis by inhibiting notch signaling [71]. Jag1 is a strong pro-angiogenic regulator that can inhibit DLL4-mediated notch signaling, as demonstrated in vitro [67]. Crosstalk with VEGF receptors (VEFGRs) is another potential mechanism through which notch signaling, directly and indirectly, controls angiogenesis [72,73]. Aberrant angiogenesis reveals the significance of notch signaling’s ability to initiate angiogenic processes. It has a significant etiological role in medical complications, including diabetes-related retinopathy, a cardiovascular disease caused by ischemia, inflammatory conditions like rheumatoid arthritis, malignant tumors, and pathological angiogenesis, which represents aberrant blood vessel formation [74]. Therefore, it may be practical to prevent pathogenic angiogenesis by targeting multiple components of the notch signaling system [75]. Notch signaling is essential for vascular stability, development, and differentiation of vascular trees by inhibiting EC proliferation and strengthening cell–cell contacts. Bone development and repair in the endochondral and intramembranous domains are governed by notch signaling, which controls cell proliferation and differentiation [76]. The notch pathway regulates VEGFR2 signaling in conjunction with CD36-like factors to generate distinct responses. Arteriolar eCs and microvascular endothelial cells (MVECs) are the primary vascular cell types that express the anti-angiogenic receptor CD36. Downregulation of anti-angiogenic genes has been associated with overexpression of CD36 [58], which alternatively leads to deficient vasculature like in tibial dyschondroplasia.

### 2.3. Ephrin-B2/EphB4 Pathway

**Figure 6:**
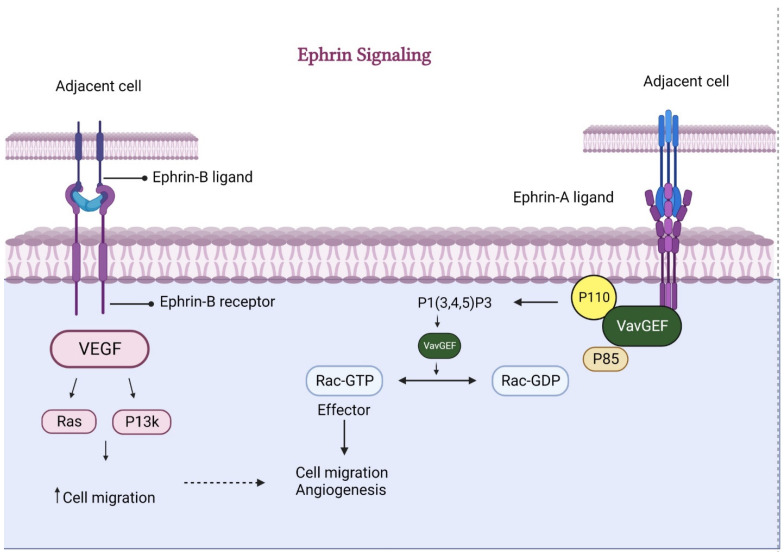
Ephrin-mediated signaling for angiogenesis. Ephrin, through both (A and B) ligands, causes increased secretion of VEGF and VavGEF, respectively. These factors consequently enhance cell migration and ultimately foster angiogenesis.

Cell contact-dependent signaling is regulated by ephrins and associated Eph receptors [77]. Ephrins primarily produce signals of repulsiveness. While EphB4 is a hallmark of venous eCs, Ephrin-B2 is present in arterial eCs. In the angiogenic endothelium, a transmembrane ligand for Eph receptor tyrosine kinases (Ephrin-B2) encourages motility and sprouting behavior [78]. Either ephrin-B2 or EphB4 deficiency causes abnormal remodeling of blood vessels [77]. For further regulation of tip cell activity and VEGFR internalization, ephrin-B2-mediated counter signaling is involved. Impaired filopodial extension and sprouting come from eCs’ inability to internalize VEGFR-2, 3 and communicate VEGF signals effectively due to a lack of Ephrin-B2 reverse signaling [78,79]. Endothelial cell migration is stimulated in response to VEGF but not fibroblast growth factor (FGF)-2, both expressed in bone. The above finding suggests that growth factor families like ephrin may modulate angiogenesis in bone and regulate the vasculature bed [80]. Chondrocytes, osteoblasts, and osteoclasts of the growth plate were expressed, while EphA4 was found in the chicken mesenchyme before chondroblast development [81]. Targeted silencing of Efnb2 or Ephb4 in mice results in poor angiogenesis, indicating the genes’ reciprocal functions [82]. The induction of the opposite signaling pathway, ephrinB2, into osteoclast precursors inhibits the development of osteoclasts and, at the same time, downregulates angiogenesis [83]. Suppression of angiogenesis in the tibial growth plate ultimately leads to TD (Figure 6).

## 3. Endogenous Anti-Angiogenic Proteins

Some naturally occurring endogenous inhibitors are also responsible for the regulation of angiogenesis. These inhibitors exhibit a diverse range of biological activity and have the potential to exert an effect on several angiogenesis-associated processes and mechanisms, such as the decreased gene expression of endothelial cells, interruption in migration and formation of eCs, or impediment in morphogenesis of the endothelial vessels in various body tissues. Because TD is characterized by simultaneous upregulation of apoptosis and downregulation of angiogenesis, the participation of endogenous anti-angiogenic proteins cannot be ruled out as a possible cause. Various factors inhibit angiogenesis, and these might play a crucial role in such conditions as shown in Table 2. 

## 4. Angiostatin

The enzymatic activity of matrix metalloproteinases (MMPs) or auto-proteolytic cleavage of plasminogen yields the polypeptide angiostatin, which consists of approximately 200 amino acids [111]. While the precise mechanism by which angiostatin suppresses angiogenesis remains unknown, this protein may cause endothelial cells to undergo apoptosis, preventing their proliferation and migration [84,112]. Similar increased apoptosis and inhibited proliferation occur in TD [52]. The suppression of MMP-mediated endothelial cell migration has also been suggested as the principal anti-angiogenic mechanism of angiostatin [113]. Angiostatin has also been hypothesized to temporarily reduce the phosphorylation of mitogen-activated protein kinases ERK-1 and ERK-2 and attenuate these proteins’ activation by pro-angiogenic growth factors like bFGF and VEGF [84].

## 5. Endostatin

One of the most powerful inhibitors of angiogenesis that are produced naturally by the body is endostatin. Endostatin is a 20-kDa C-terminal fragment of type XVIII collagen [114]. Endostatin is responsible for the downregulation of signaling pathways in the microvascular endothelium that modulate angiogenic factors such as VEGF, bFGF, HIF-1 hepatocyte growth factor (HGF), and tumor necrosis factor (TNF) receptor, as well as the upregulation of many genes that encode for anti-angiogenesis, e.g., thrombospondin-1, HIF-1-inhibitor, and maspin [115]. Endostatin inhibits cell movement and triggers programmed cell death in endothelial cells [116,117]. It also inhibits angiogenesis by preventing bFGF and VEGF through their pro-angiogenic roles [118]. Endostatin blocks FGF signaling, preventing the cytoskeleton’s remodeling and the endothelial cells’ adherence to the extracellular matrix [119]. This ultimately leads to control over apoptosis in endothelial cells. Ameliorated symptoms of TNF-induced inflammatory arthritis in mice were treated with endostatin by decreasing vascular density in synovial tissues [120]. Its anti-angiogenic role makes endostatin a subject of concern regarding TD.

## 6. Vascular Endothelial Growth Inhibitors

The anti-angiogenic cytokine vascular endothelial growth inhibitor (VEGI) belongs to the tumor necrosis factor (TNF) superfamily [121]. Isoforms of VEGI include VEGI-174, VEGI-192, and VEGI-251. VEGI has the ability to activate various signaling pathways, such as p38 mitogen-activated protein kinase, nuclear factor-kappa B, and c-Jun N-terminal kinase. In addition, it inhibits tumor growth, endothelial cell proliferation, and angiopoiesis [122]. Due to its role as an autocrine factor, it causes endothelial cell apoptosis and stops their growth both in vitro and in vivo [123]; similar large-scale apoptosis found in TD, where the role of such growth inhibitors requires investigation.

## 7. Decoy Receptors

A “decoy receptor” is a protein that binds to particular growth factors or cytokines but cannot transmit a signal. These proteins have been hypothesized to function as blockers in various signaling cascades. Endothelial cells express vascular endothelial growth factor receptor 1 (VEGFR-1), also known as fibronectin receptor 1 (FLT-1). VEGFR-1 is a highly sensitive receptor for vascular endothelial growth factors A and B [124]. VEGFR-1′s precise function in angiogenesis is unknown; nonetheless, its loss causes excessive endothelial cell proliferation and developing embryo death [125]. Soluble VEGFR-1 functions as a decoy receptor, potentially attenuating angiogenesis. In order to achieve typical development and angiogenesis, VEGFR-1 must retain its decoy property. VEGFR-1 reduces VEGFR-2 activity by appropriating and trapping VEGF, which prevents VEGFR-2 from binding to VEGF [126,127]. There is an increase in VEGF-A in TD but no corresponding rise in vasculature [52], which may be due to decoy receptors but requires some investigation.

Neuropilin (NRP1) is a further example of a decoy receptor that blocks angiogenesis. NRP1 is a membrane-bound coreceptor associated with a tyrosine kinase receptor for VEGF isoforms, specifically VEGF165 [128]. NRP1 mediates various critical cellular processes, such as axon guidance [129], cell survival, migration, and invasion [130]. Angiogenesis mediated by VEGF is regulated by the nuclear receptor protein 1 (NRP1) [128,131]. Endothelial cells with high levels of NRP1 expression have increased chemotaxis and mitogenesis because VEGF165 has a higher affinity for the receptor VEGFR-2. In contrast to membrane-bound NRP1, the soluble version of neuropilin (sNRP1) possesses anti-VEGF characteristics [132]. It has been shown that sNRP1, a 90-kDa protein, binds with VEGF165 and prevents both the protein’s attachment to endothelial cells and the tyrosine phosphorylation of KDR. Additionally, it was shown that sNRP-1 injections might stop the development of acute myeloid leukemia in mice in vivo, which means sNRP1 prevents VEGF-mediated angiogenesis [132].

## 8. Anti-Angiogenic Chemokines and Chemokine Receptors

Within this class of proteins, in addition to pro-angiogenic chemokines, there are also some anti-angiogenic factors. CXC chemokines devoid of the ELR motif, often known as “CXC ELR chemokines,” are typically chemo-attractive to T and NK cells in lymphocytes [133]. Its chemokine members bind primarily to the chemokine receptor CXCR3 that is normally found on T lymphocytes, as well as certain B and NK cells and some endothelial cells [134]. It has been revealed that the chemokine receptor CXCR3 can play an important role in mediating the angiostatic activity of ELR chemokines [135,136]. Chemokines that bind to CXCR3, such as CXCL4, CXCL9, CXCL10, and CXCL11, may inhibit growth factor-induced endothelial cell chemotaxis and vessel formation. Via a positive feedback loop, CXC ELR (−) chemokines may boost the recruitment of NK and Th1 cells, preventing angiogenesis [137]. Furthermore, these chemokines caused blood vessel loss in vivo and regressed newly generated cords in vitro.

CXCL10 chemokine was found to have an additional angiostatic action that might lead to the separation and regression of newly formed blood vessels in wound healing and also the death of endothelial cells [138]. Apoptosis or regression of vessels may result from attaching angiostatic chemokines with respective receptors on endothelial cells. These chemokines may also play a role in TD’s large-scale apoptosis and vascular necrosis [139]. The attachment and deceiving of pro-angiogenic growth factors through ELR can also contribute to its anti-angiogenic activity. According to research conducted by Rath et al., vascular endothelial growth factor (VEGF) receptor disruption leads to endothelial cell death, which in turn impedes vascularization, the clearance of dead chondrocyte arteries, and the regression of damaged tissue during the wound-healing process [19], which may also be correlated with anti-angiogenic chemokine activity.

## 9. Vasoinhibins

**Figure 7:**
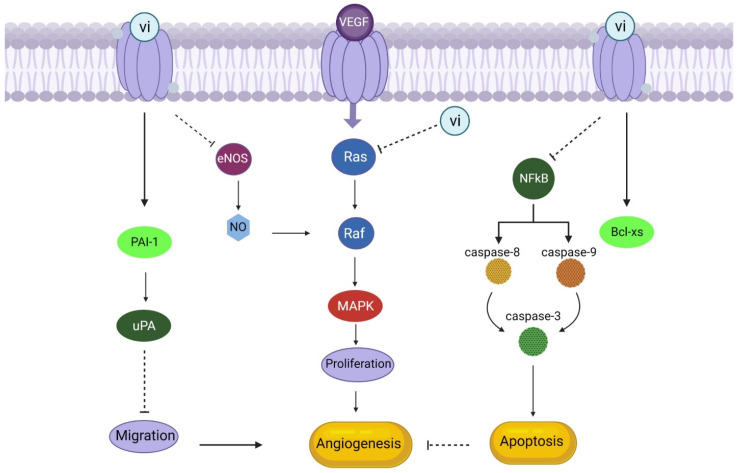
Vasoinhibins as an anti-angiogenic factor (Vasoinhibin (vi) binds with its receptors, stimulates apoptosis, and inhibits migration through upregulation of Bcl-xs and PAI-1, respectively. Moreover, it can inhibit the activation of eNOS and MAPK pathways activated by VEGF, ultimately resulting in the downregulation of angiogenesis).

The vasoinhibin family of peptides includes prolactin, growth hormone, and placental lactogen that acts on endothelial cells to reduce vasodilation and angiogenesis while increasing apoptosis-mediated vascular regression. By interfering with growth factor activity, suppressing protease synthesis, and inducing apoptosis in endothelial cells, vasoinhibins can prevent multiple phases in the angiogenic process (Figure 7). In vivo [140,141,142,143] and in vitro, vasoinhibins inhibit angiogenesis by preventing vascular endothelial growth factor (VEGF)-induced vasodilation of chondrocyte vessels and their regression during wound healing [144]. VEGF and bFGF both stimulate endothelial cell proliferation [145], and basic fibroblast growth factor (bFGF) increases urokinase plasminogen activator (uPA) activity [141,146]. Moreover, vasoinhibins can prevent the formation of capillary-like structures in collagen gels by inhibiting the basal activity of uPA by promoting the expression of plasminogen activator inhibitor type 1 (PAI-1) [140,145]. Regression of blood arteries is mediated by apoptosis, and vasoinhibins can trigger apoptosis of endothelial cells in vitro [146]. Even in the absence of growth stimuli [147,148], there is a possibility that vasoinhibins can inhibit angiogenesis in specific tissues. Chondrocytes derived from articular cartilage are capable of producing PRL as well as vasoinhibins, and they exhibit a higher potential for vasoinhibin production compared to other cell types [149], but the anti-angiogenic role of vasoinhibins has not yet been evaluated in cartilage which needs to be evaluated like in tibial dyschondroplasia in chickens.

## 10. Signal Transduction of Vasoinhibins as Anti-Angiogenic Factor

Vasoinhibins suppress cyclins D1 and B1, which stop cell division from occurring throughout the G0-G1 and G2-M phases of the endothelial cell cycle [150]. This may be because vasoinhibins prevent Ras-dependent activation of the MAPK pathway in response to vascular endothelial growth factor (VEGF) and basic fibroblast growth factor (bFGF) [151,152]. The activity of endothelial nitric oxide synthase (eNOS) elicited by VEGF was demonstrated to be blocked by vasoinhibins, and exogenous restored the suppression of VEGF-driven endothelial cell proliferation by vasoinhibins (109 [144]. NO interacts with the MAPK pathway through a cascade that includes NO promotion of cGMP synthesis and activation of cGMP-dependent protein kinase (PKG), ultimately leading to RAF activation to contribute to the mitogenic effect of VEGF [153]. Thus, vasoinhibins can inhibit MAPK activation by blocking RAS and PKG.

While NO produced by eNOS enhances blood artery permeability and hinders the adherence of leukocytes and platelets to endothelial cells, blocking eNOS activity with vasoinhibins also decreases vasodilation [144]. This may have further vascular effects. Since Ca^2+^-calmodulin (Ca^2+^-CaM) binding is necessary for eNOS activation, and vasoinhibins block acetylcholine- and bradykinin-induced Ca^2+^ transients in endothelial cells (Figure 7), it is possible that vasoinhibins interfere with the mobilisation of intracellular Ca^2+^ [154]. The mechanism by which vasoinhibins block eNOS activation is unknown [144]. In addition, vasoinhibins suppress interleukin 1 (IL-1)-induced inducible nitric oxide synthase (iNOS) expression, which inhibits NO generation by endothelial cells [155]. Vasoinhibins may counteract IL-1′s anti-tumor angiogenesis activity by inhibiting the MAPK-signal transducer and activator of transcription 1-interferon regulatory factor 1 pathway [155]. The breakdown of the NF-kB inhibitor and the consequent stimulation of caspases 8, 9, and 3 via the extrinsic and intrinsic apoptotic pathways suggest that vasoinhibins drive apoptosis. Furthermore, vasoinhibins can trigger the switch from Bcl-XL to Bcl-XS, the proapoptotic form of Bcl-XL [156].

## 11. Thrombospondin

Bouck and colleagues identified TSP-1 as the first protein augmented by tumor suppressor genes acting as a natural angiogenesis regulator. TSP-1, a heparin-binding protein found in the extracellular matrix, appears to disrupt connections between endothelial cells and suppress the proliferation of endothelial cells from various tissues [85]. The N-terminal heparin-binding domain of the protein contains the active site responsible for the anti-angiogenic action. [157]. Using various assays, the angiostatic and angiotoxic effects of TSP-1 have been reported [158], deleting TSP-1 in mice entirely blocked the anti-angiogenic therapy’s anticancer effect, and anti-angiogenic chemotherapy raised circulating TPS-1. Several studies have investigated TSP-1 for potential therapeutic uses due to its role as a powerful endogenous angiogenesis inhibitor.

An 80-kDa fragment in the N-terminal region of TSP-2 reduces endothelial cell migration and vessel formation while simultaneously boosts endothelial cell-specific programmed death [159]. TSPs have been shown to have either pro-angiogenic or biphasic functions [160,161]. These results align with the theory that TSPs are multicellular, with distinct domains of the proteins interacting with different receptors of endothelial cells to elicit highly varied responses and operate as natural anti-angiogenic agents [162].

## 12. Endogenous Inhibitors of Metalloproteinases

Four tissue inhibitors of metalloproteinases (TIMPs) are among the several endogenous inhibitors that regulate MMP activity in the extracellular milieu. TIMP-3 attaches to the extracellular matrix (ECM), whereas TIMP-1, -2, and -4 are soluble [163]. TIMPs should have an anti-angiogenic effect because they are critical regulators of MMP activity. For instance, the anti-angiogenic activity of cartilage was thought to be due to the high concentration of TIMPs in cartilage extract [164]. As TD is most relevant with cartilage through the growth plate and then conversion into bone, inhibitors of metalloproteinases are a subject of interest.

## 13. PEX

Matrix metalloproteinase MMP-2 has a non-catalytic C-terminal hemopexin-like domain (PEX) that can interact with integrin avb3 to prevent MMP-2 binding and subsequent angiogenesis. MMPs are tri-domain proteins that have a pro-domain at their N-termini, a catalytic Zn2+ protease domain in the middle, and a PEX hemopexin-like domain at their C-termini. It seems that PEX2 results from the breakdown of MMP-2. As a major macromolecular constituent of basement membrane, type IV collagen is thought to play an important role in EC proliferation and behavior during the angiogenic process, and this protein fragment can inhibit cell-associated collagenolytic activity with preferential substrate specificity towards type IV collagen [165]. Therefore, PEX regulates healthy neovascularization and angiogenesis by suppressing their respective processes. PEX binding to avb3 is RGD-independent since MMP-2 lacks the sequence. The PEX domain of MMP9 is an asymmetric, non-covalent homodimer made up of several b-sheets. PEX9 has a b-propeller shape with four rotor blades, and blade IV mediates non-covalent, hydrophobic dimerization interaction between basement membranes [166].

## 14. Troponin I

Troponin I (Tn I) is a 24 kDa protein produced from cartilage and acts as an angiogenesis inhibitor by preventing endothelial cell proliferation and new blood vessel formation in in vitro and in vivo settings [167]. By interacting with the FGF-2 receptor on the surface of endothelial cells, Tn I suppresses proliferation in both activated and non-activated endothelial cells [107]. So, troponin I overexpression might lead to deficient vascularization and associated diseases in cartilage and growth plates.

## 15. Conclusions

Tibial dyschondroplasia is among the most devastating bone diseases of fast-growing poultry birds. The pathogenesis of the disease involves reduced angiogenesis and rapid apoptosis with compromised chondrocyte viability at the hypertrophic zone of the growth plate, ultimately leading to poor osteogenesis through HIF-1α/VEGF/VEGFR signaling pathways. The underlying mediators of these pathways are multifactorial and require a proper balance for physiological growth. Hence, there is a gap in research regarding the strict balance between pro and anti-angiogenic growth factors and cytokines that could be an interesting field of study for researchers to determine new therapeutic approaches for such bone disorders.

## Figures and Tables

**Table 1:** Important vascular growth regulation factors.

**Mediators**	**Process**	**Function**
VEGF		Angiogenesis stimulation and permeability elevation
VEGF-C		Lymphangiogenesis stimulation
PlGF		Angiogenesis in pathological conditions
Angiopoietin-1		Vascular stabilization through improving smooth muscle and endothelial cell interactions
PDGF	Angiogenesis	Stimulate smooth muscle cell recruitment
TGF		Stabilizes blood vessels by stimulating ECM
FGF		Atherogenesis and angiogenesis stimulation
HGF		Angiogenesis stimulation
MCP-1		Stimulation of atherogenesis
Ephrins		Cause differentiation between the artery and vein
Metalloproteinase		Matrix remodeling and cell migration, as well as stimulation and plasminogen activators of FGF&VEGF
Cyclooxygenase and NO synthase		Vasodilation and angiogenesis stimulation
Inhibitors		Function
Angiostatin		Inhibits endothelial migration as well as cell survival
Angiopoietin-2		Regression and destabilization of blood vessels in the absence of angiogenic factors
Interleukin and Interferons		Inhibits endothelial migration
Endostatin		Reduces cell survival and endothelial migration
Endostatin		Vascular growth inhibition

**Table 2:** Endogenous inhibitory factors of angiogenesis.

**Anti-Angiogenic Factor**	**Mechanism**	**Reference**
Angiostatin	Epithelial cells apoptosis	[84]
Endostatin	Decreases VEGF, bFGF, and TNF	[85]
Arresten	MAPK/MEK/c-Raf pathways	[86]
Canstatin	Promote apoptosis	[87]
Endorepellin	Integrin α2β1pathway of angiostatic activity	[88]
Fibronectin fragment	Erk pathway	[89]
Vasohibin	Vascular endothelial growth factor activity	[90]
Interferons IFNA1,2, etc.	CXCL-10 activity	[91]
Fibulin	Erk pathway	[92]
Thrombospondin	Erk/rac pathway and Phosphoinositide 3-kinase	[93,94]
Tumstatin	Akt/mTOR signaling	[95]
Pentraxin	FGF2-mediated pathway	[96]
Pigment epithelium derived factor (PEDF)	VEGF-mediated signaling	[97,98]
Anti-thrombin III	NFkB/ perlecan pathway	[99,100]
Platelet factor IV	VEGF and FGF2 signaling	[101]
Tissue inhibitors of metalloproteinases (TIMPs) 1–4	MMPs-mediated pathways	[102]
PEX	Matrix metalloproteinase-2	[103]
Vasostatin	TNFα and MAPK-mediated gap formation	[104]
Angiopoietin-2	Antagonize the binding Tie2 via angiopoietin-1	[105]
Tyrosine kinase-1	VEGF pathway	[106]
Troponin I	Basic fibroblast growth factor (bFGF)	[107]
Somatostatin	MAPK and nitric oxide synthase pathway	[108]
Interleukin 1, 4, 12, 18	Nitric oxide, vascular permeability factor, VEGF, MMPs, TBS1, and CXCL8,9,10 activity	[109,110]

## Data Availability

Not applicable.
